# Bilateral disease and intratesticular haemodynamics as markers of dyspermia in patients with subclinical varicocele: A prospective study

**DOI:** 10.1080/2090598X.2019.1647676

**Published:** 2019-08-01

**Authors:** Georgios Tsampoukas, Athanasios Dellis, Athanasios Papatsoris

**Affiliations:** a Urologist, General Hospital of Patras, Patras, Greece; b Urology, National and Kapodistrian University of Athens, Aretaieion Academic Hospital, Athens, Greece; c Urology, National and Kapodistrian University of Athens, ‘Sismanoglio’ General Hospital, Maroussi, Athens, Greece

**Keywords:** Subclinical varicocele, ultrasound, bilateral varicocele, EDV, RI, semen parameters

## Abstract

**Objective**: To study scrotal ultrasonographic characteristics in patients with subclinical varicocele (SV) and investigate their relationship with semen parameters.

**Patients and methods**: In all, 56 men with SV were recruited and divided into two groups, according to their semen characteristics. Group A, comprised 34 men with normal semen analysis; and Group B, comprised 22 men who carried at least one abnormality, regarding sperm concentration, motility and morphology. Between the two groups we compared: age; body mass index (BMI); semen pH and semen volume; total testicular volume (TTV); maximal vein diameter (MVD) and degree of reflux; mean values of peak-systolic velocity (PSV), end-diastolic velocity (EDV), and resistive index (RI) of the intratesticular arteries; whether bilateral SV; and serum testosterone and follicle-stimulating hormone (FSH) levels.

**Results**: Asthenospermia was present in all patients in Group B; 10 patients had asthenospermia only, six patients were astheno-oligospermic and six patients had astheno-oligo-teratospermia. Age, BMI, semen pH and volume, TTV, MVD and degree of reflux did not differ significantly between the two groups (*P* > 0.05). However, EDV, PSV and RI were significantly different (*P* < 0.05). Bilateral SV was significantly more frequent in patients in Group B (*P* < 0.05). Finally, FSH was elevated in Group B (*P* < 0.05), whereas testosterone was normal in both groups, albeit significantly lower in men with abnormal semen analyses (*P* < 0.05).

**Conclusion**: Classic ultrasonographic characteristics in men with SV, such as venous size or degree of reflux, were insufficient to distinguish patients with abnormal semen analysis. However, bilateral disease and intratesticular haemodynamics differed significantly in patients with SV and abnormal semen analysis.

**Abbreviations**: BMI: body mass index; CDU: colour Doppler ultrasonography; EDV: end-diastolic velocity; MVD: maximal vein diameter; PSV: peak-systolic velocity; RI: resistive index; SV: subclinical varicocele; TTV: total testicular volume; US: ultrasonography

## Introduction

Subclinical varicocele (SV) is defined as the abnormal dilation of the veins of the pampiniform plexus, not clinically detectable but diagnosed by imaging modalities [1]. Although venography is considered more sensitive, its invasiveness renders colour Doppler ultrasonography (CDU) as the more widespread tool for diagnosis [2]. Also, ultrasonography (US) can assist in long-term surveillance, especially in children and adolescents [3]. The significance of SV remains a huge debate, as far as cumulative conclusions from randomised trials have not shown a clear benefit of correction on pregnancy rates [4,5]. However, findings indicate that the condition is involved in the pathophysiological pathway that results in hypoxia and testicular dysfunction [6]. Also, the condition should be considered a dynamic phenomenon, as nearly 28% of children with SV will develop clinical varicocele over a period of 4 years [7], and physical activity increases the risk of progression [8]. Furthermore, a right SV is found in up to 57.8% of infertile patients with left clinical varicocele, and thus, the condition should not be disregarded [9]. Additionally, cumulative data highlight the superiority of bilateral varicocelectomy regarding higher pregnancy rates, in cases of concurrent right SV and left clinical varicocele compared to a unilateral procedure [10]. Therefore, SV may have a role in infertility. As no specific criteria exist that distinguish patients at risk, the objective of the present study was to investigate the significance of scrotal US, a routine diagnostic modality in daily urological practice, in the assessment of the impact of SV on semen parameters.

## Patients and methods

Our prospective study was conducted at the urological departments of two hospitals between May 2017 and June 2018. Inclusion criteria were the presence of SV, unilateral or bilateral. Patients with clinical varicocele, systemic disease, history of cancer, chronic or active accessory gland infection, cryptorchidism, previous inguinal surgery, signs of obstruction within the genital tract, testosterone supplements intake, and alcohol or other drug abuse, were excluded from the study. The recruitment process included patients seeking consultation in our clinics for infertility issues or men who presented for other reasons to our department and who were informed about the study. The study was approved by the Scientific Boards of both institutions and the patients were informed about the study, giving written consent. Physical examination was performed in a warm room by two separate urologists, in a standing position. A US examination was performed using a 12-MHz probe in a standing position during normal respiration and during a Valsalva manoeuvre. The diagnostic criteria of a SV were the absence of a clinical varicocele in the physical examination and the presence of dilated veins in the pampiniform plexus of >2 mm, demonstrating reflux during the Valsalva manoeuvre on CDU. The reflux was graded using the Hirsh et al. [11] classification, which divides reflux according to its spontaneity into three grades. Grade I is defined as a Valsalva-induced reflux in the dilated veins and is subdivided into two patterns: pattern I, defined as reflux inducible during Valsalva and fading out before the end of the manoeuver; and pattern II reflux, detectable during the whole duration of the manoeuver. Grade II and III are defined as spontaneous reflux demonstrated intermittently or constantly, respectively. The testicular volume (TV) was measured automatically by the US unit according to the formula: volume = 0.53 × length × width × height. Then, semen sample analysis according to WHO criteria was requested. In cases of a normal semen analysis, one analysis was considered sufficient. In cases of an initial abnormal result, a second analysis was requested for confirmation. When severe oligospermia was detected (<1 million spermatozoa/mL), a karyotype and evaluation for azoospermia factor (AZF) was requested. Patients with abnormal results were excluded from the study. Subsequently, Group A comprised all patients with normal semen parameters and Group B patients with at least one abnormal semen parameter regarding concentration (oligospermia defined as <15 millions of spermatozoa/mL), motility (asthenospermia defined as <32% progressive motility) and morphology (<4% normal forms), according to current guidelines [12]. The parameters for comparison included: age; body mass index (BMI); semen volume and pH; total TV (TTV); mean peak-systolic velocity (PSV), mean end-diastolic velocity (EDV), mean resistive index (RI) in the intratesticular arteries; unilateral/bilateral SV; FSH and serum testosterone levels; and maximal vein diameter (MVD) on both sides. For statistical analysis, the Shapiro–Wilk test was used to check normality and subsequently, the Student’s *t*-test, Mann–Whitney *U*-test and chi-squared test were used accordingly for the detection of statistically significant differences in parameters between the two groups. Statistical significance was set at a *P* < 0.05.

## Results

In all, 56 men were included in the study; 34 with SV who had normal semen parameters (Group A) and 22 with SV and abnormal semen parameters (Group B). In the latter group, 10 patients had asthenospermia only, six had astheno-oligospermia and six had astheno-oligo-teratospermia. Age, BMI, MVD on the left and right side, semen pH and volume, and TTV did not differ between the groups. On the contrary, the mean RI was significantly different between the groups (0.51 for Group A vs 0.57 for Group B, *P* = 0.01). Also, the mean PSV (10.01 vs 8.51 cm/s, *P* = 0.042) and mean EDV (4.76 vs 3.57 cm/s, *P* < 0.05) differed significantly between the groups. Also, FSH was markedly elevated in Group B, whereas serum testosterone was within normal limits, but was significantly lower in Group B (*P* < 0.05). The patients’ characteristics are listed in Table 1. Furthermore, all men in our study had a left SV, whereas a bilateral SV was detected in four of the 34 patients in Group A and in 12 of the 22 in Group B (11.8% vs 54.5%, *P* = 0.02; Table 2). Regarding reflux, all SVs were Grade I; in Group A 58.8% of the left SVs were pattern 2, whereas in Group B 72.7% were pattern 2, although the difference was not significant. On the right side, most of the SVs had pattern 1 reflux (three of four in Group A, eight of nine in Group B) and again the groups did not differ significantly (Table 3).

**Table 1.** Patients’ characteristics.

Characteristic	Group	*Ν*	Mean (SD)	*t*	*U*	*P*
Age, years	Α	34	25.26 (4.56)	–1.217		0.229
	Β	22	26.82 (4.83)			
Mean RI	Α	34	0.51 (0.06)	–3.655		0.001*
	Β	22	0.57 (0.06)			
Left MVD, mm	Α	34	3.15 (0.37)	–1.176		0.245
	Β	22	3.29 (0.53)			
Serum testosterone, ng/dL	Α	34	530.00 (90.90)	2.236		0.030*
	Β	22	469.09 (111.82)			
BMI, kg/m^2^	Α	34	23.66 (2.86)		303.5	0.237
	Β	22	22.72 (2.98)			
Right MVD, mm	Α	3	3.05 (0.78)		21	0.900
	Β	13	2.93 (0.42)			
TTV, mL	Α	34	28.81 (5.35)		281.5	0.121
	Β	22	27.15 (9.07)			
PSV, cm/s	Α	34	10.01 (2.51)		252.5	0.042*
	Β	22	8.51 (2.01)			
EDV, cm/s	Α	34	4.76 (1.01)		133	<0.001*
	Β	22	3.57 (0.89)			
Semen pH	Α	34	7.61 (0.32)		408	0.566
	Β	22	7.65 (0.36)			
Semen volume, mL	Α	34	3.97 (1.20)		300	0.204
	Β	22	3.59 (1.08)			
FSH, mUI/mL	Α	34	2.74 (0.90)		664	<0.001*
	Β	22	6.69 (4.59)			

*Statistically significant difference, *P* < 0.05.

**Table 2.** Presence of right SV, i.e., patients with bilateral SVs.

	Right varicocele		
	Absent	Present	Overall
Group Α, *n* (%)	30 (88.2)	4 (11.8)	34 (100)
Group Β, *n* (%)	10 (45.5)	12 (54.5)	22 (100)
Overall, *n* (%)	40 (71.4)	16 (28.6)	56 (100)

Chi-squared = 9.974, d.f. = 1, *P* = 0.02

**Table 3.** The patients’ Grade I reflux classifications for left- and right-sided SVs.

	Reflux left-side*			Reflux right-side**		
	Pattern I	Pattern II	Overall	Pattern I	Pattern II	Overall
Group Α, *n* (%) or *n/N*	14 (41.2)	20 (58.8)	34 (100.0)	3/4	1/4	4/4
Group Β, *n* (%) or *n/N*	6 (27.3)	16 (72.7)	22 (100.0)	8/9	1/9	9/9
Overall, *n* (%) or *n/N*	20 (35.7)	36 (64.3)	56 (100.0)	11/13	2/13	13/13

*Chi-squared = 0.601, d.f. = 1, *P* = 0.438; **Chi-squared = 0.410, d.f. = 1, *P* = 0.522

## Discussion

In our present study, it was difficult to set a specific vein size diameter criterion for the diagnosis of SV, as there is great discrepancy amongst studies regarding the optimal threshold [3]. Previously, a diameter of ≥3 mm has been used as a criterion for the diagnosis of SV [13,14], whereas another widespread threshold was set at 2.7 mm by Jarow et al. [15]. The changeover of a SV to a clinical varicocele seems likely as venous size increases; Hoekstra et al. [16] concluded that a clinical varicocele is unlikely not to be found above the level of 3–3.5 mm. However, in the range of 3 to 4 mm, Metin et al. [17] reported that palpable varicoceles can be found in 50% of cases, whereas the upper limit of vein diameter in the pampiniform plexus of normal subjects has been found to be up to 3.8 mm [18]. Furthermore, when comparing US measurements and actual measurements during varicocelectomy, a venous size overlapping between SVs and clinical grades is commonly found [19]. Thus, it seems rational, that neither a lower value nor upper limit exists to dictate the diagnosis of a SV. This is the reason why widespread classification systems for varicocele in general are based on a combination of other characteristics rather than diameter solely. For instance, Chiou et al. [20] proposed a system based on the combination of MVD and sum diameter, and duration of reflux; whilst other classification systems, e.g., by Hirsh et al. [11], Liguori et al. [21] or the Sarteschi’s classification do not take into consideration the diameter of the veins, being based mainly on the severity of the reflux and the condition of the testes. Therefore, in our present study, in cases of a negative physical examination, we made a diagnosis of SV when dilated veins were >2 mm in diameter and exhibiting retrograde flow during the Valsalva manoeuvre, which is considered the lower threshold proposed by Gonda et al. [22]. Then, we relied on the subsequent statistical analysis to give prominence to the significance of MVD, without setting an upper limit. According to our observations, there was no correlation between MVD and the presence of abnormal semen parameters; men in Group B had larger SVs on the left side, but the difference was not significant; on the right side, men in Group B had non-significantly less dilated veins than those in Group A. So, in our present cohort, the conclusion was that venous size corresponding to MVD was not a decisive criterion to predict dyspermia in men with SV. These findings resemble those of previous reports, in that preoperative venous size measurement is not a reliable indicator regarding outcome of varicocelectomy [19]. Similarly, no significant difference in postoperative paternity rates has been reported for varicoceles >4 mm in comparison to those that are less dilated [23]. Moreover, Chen [24] reported no significant difference in MVD between patients with SV and normal and abnormal semen parameters. Last but not least, in another study, SVs which tend to gain so called ‘clinical relevance’, i.e., >3 mm, are associated with greater improvement after varicocelectomy [15].

For reflux, we stratified our present patients according to the Hirsh classification system [11]. Use of this system is widespread and it carries significance as far as increasing reflux grade is associated with deterioration in semen parameters [25]. In our present study, all cases were classified as Grade I, viz. no spontaneous reflux was observed; our findings are in accordance to the literature, as far as this grade is consistent with SV [7,26]. Also, we examined the significance of the subdivisions, patterns 1 and 2. Pattern 1 is similar to stop-type reflux and indicates competence of the valves as far as retrograde flow fades out before the end of the Valsalva manoeuver [23]. Pattern 2, equivalent to shunt-type reflux, implicates some degree of weakness of the spermatic vein valves or secondary venous communications and it is associated with progression of testicular asymmetry if left untreated and has a higher risk of recurrence [27]. Also, pattern 2 combined with any degree of clinical varicocele puts adolescents at risk of testicular hypotrophy [28]. In our present study, there was no significant difference between the two groups for the type of reflux; however, men with SV and abnormal semen parameters had pattern 2 at higher frequency (72.7% vs 58.8%, *P* > 0.05). Although the difference was not significant, such a venous abnormality could be a prerequisite for the manifestation of dyspermia in the patients with SV, but this hypothesis needs further investigation.

Comparing age, there was no difference between the two groups for the risk of abnormalities in semen parameters. However, reports in the literature implicate age in the prognosis after treatment. For instance, treating Grade I varicocele (according to Hirsh varicocele) in men aged >30 years was associated with poor improvements in both semen quality and pregnancy rates [29]. In another study, Shiraishi et al. [19] reported that in patients aged <30 years there was no significant difference between spermatic vein diameter in patients with improved outcome and those with unchanged or worsened outcome, whereas in patients aged >30 years, larger vein diameters were associated with suboptimal results.

The somatometric traits of our present patients via BMI were also compared between the two groups and no significant difference was noted. In general, a strong correlation applies between BMI and varicocele, as taller, non-obese men with lower BMIs are more likely to be diagnosed with clinical varicocele [30,31]. Such observations are aligned with the hypothesis that the manifestation of the condition is the result of hydrostatic pressures within the veins, which depends heavily on the vertical column of the internal spermatic veins [6]. However, BMI cannot be used as a predictor regarding semen quality [25,32], nor can it predict grades of varicocele [31]. In the setting of a SV, Chen [24] reported that BMI cannot differentiate patients with SV who might be at risk of subfertility; in addition, BMI did not differ significantly between patients with SV and normal men.

Although men in Group B had lower TTVs, the difference was not significant. These findings indicate that TV is not the issue in subfertile men with SV, but some further findings are noteworthy. Previously, Chen [24] reported that men with SV and a TTV <27 mL are likely to be subfertile. Using that threshold, 13 of the 22 patients (59.1%) in our cohort with a TTV <27 mL carried at least on semen abnormality, whereas only nine of 34 (26.5%) patients with a TTV >27 mL had abnormal semen samples, a significant difference (*P* < 0.05). Moreover, conclusions from a study of adolescents with clinical varicocele indicate that a TTV of <30 mL, quadruples the risk of a low total motile sperm count [33]. In our present study, 16/40 patients (40%) with a TTV <30 mL had abnormal semen analysis, a percentage almost equal to men with a TTV >30 mL (six of 16, 37.5%), a non-significant difference (*P* > 0.05). So, lower TVs may place patients with SV at risk of an impaired semen analysis. Regarding the effect of SV on ipsilateral testicular growth, discrepancies amongst studies indicate that the role of the condition in testicular asymmetry is rather inconclusive [13,34]. In our present cohort, we did not examine the impact of SV on ipsilateral growth, as the number of participants was limited, we did not have a control arm of normal men, and also a significant number of participants had bilateral disease.

Also, we investigated the association of intratesticular haemodynamics and dyspermia in men with SV. Men in Group B were found to have significantly lower mean EDV and PSV values than men in Group A. Our measurements are consistent with reports that implicate alterations in testicular blood flow in the pathogenesis of clinical varicocele and they indicate that even SV may affect testicular microcirculation and subsequently, impair spermatogenesis. Tarhan et al. [35] reported that clinical varicocele is associated with decreased testicular blood flow compared with healthy individuals and the authors advocated that hypoperfusion may negatively affect spermatogenesis. Similarly, in an experimental study, a decrease in testicular blood flow was observed after the induction of a left varicocele [36]. On the other hand, Ross et al. [37] have observed that neither varicocele nor varicocelectomy alter testicular blood flow in men, whereas experimental data demonstrated that varicocele increased testicular blood flow, which might be the reason for testicular hyperthermia [38,39]. Used as markers, Ener et al. [40] reported that both EDV and PSV were increased significantly after varicocelectomy, indicating a restoration of testicular microcirculation, whereas, in another study, elevation of EDV after varicocelectomy in the affected side was associated with a successful outcome [41]. In our present study, decreased EDV values of patients in Group B may correspond to clinically meaningful impedance of blood flow inside the testicular parenchyma. Similarly, decreased PSV values may reflect some degree of hypo-perfusion. Both phenomena may be associated with impaired microcirculation and subsequent impaired spermatogenesis in patients with SV.

Our present study showed that the RI might have a role in the assessment of dyspermia in men with SV; patients in Group B had significantly higher mean RI values. According to previous reports, Pinggera et al. [42] and Hillelsohn et al. [43] have indicated the RI as a marker of dyspermia, as values >0.6 are strongly associated with abnormal semen parameters. In correlation with clinical varicocele, a decrease in intratesticular RI after varicocelectomy has been correlated positively with improvement in semen parameters [44]. In cases of SV, Chen [24] reported that RI values >0.55 are strongly associated with subfertility, results that are consistent with the findings of our present study. Finally, Akcar et al. [45] reported no impact of SV on ipsilateral testicular RI of infertile men; however, patients in that study were all infertile and RI values were also elevated (0.61 for left side, 0.58 for right side, in both the varicocele and control groups). In our present study, we found that the RI could distinguish those men with SV who may be at risk of subfertility. However, the optimal threshold needs further investigation.

There was a statistically significant difference in the presence of bilateral SVs between the two groups, with most of the men in Group A having unilateral SV, whereas most of the men in Group B had bilateral SVs. Our findings should be considered meaningful, as patients with bilateral SVs seem to have a greater likelihood of having abnormal semen parameters. Our present observations are in accordance with the literature concerning the role of bilateral varicocele regardless of size. Firstly, according to Trussell et al. [9], up to 77.5% of infertile men with varicocele may have bilateral disease. In cases of a concurrent left clinical varicocele and right SV, bilateral varicocelectomy has been found superior to unilateral varicocelectomy for both improvement in semen parameters and increase in pregnancy rates [10]. Other anatomical aspects should also be taken into consideration, which attach extra importance to bilateral disease; Sakamoto and Ogawa [46] found that bilateral, clinical varicoceles and SVs, are much more frequently correlated with dilation of the prostatic venous plexus in comparison to unilateral SVs or no disease. Furthermore, in a study of men with bilateral clinical varicoceles with asthenospermia, the simultaneous presence of a dilated prostatic venous plexus was accompanied strongly with hyperviscosity; patients with a dilated prostatic venous plexus did not have a significant improvement in their motility and viscosity after varicocelectomy compared to those who did not have a dilated prostatic venous plexus [47]. Finally, an interesting finding was reported by Cervellione et al. [7], who observed that none of the children with bilateral SV developed a clinical varicocele during a long-term follow-up.

FSH was found to be markedly elevated in men in Group B, an observation that was expected due to the deterioration in semen parameters in this group. Semen volume and pH were included in the comparison between the two groups in order to investigate if, in the absence of other pathology, semen abnormalities in men with SV were accompanied with changes in these parameters that could implicate impairment of the male accessory glands. Semen volume did not differ significantly between the groups and our observations were similar with previous reports showing that clinical varicocele has limited impact on semen volume [48]. Similarly, having previously excluded conditions that could dramatically alter the acidity of the seminal fluid, semen pH did not differ between the two groups either [1]. Importantly, we did not find a conclusive impact of SV on endocrine testicular function, as serum testosterone was found to be within normal limits in all patients but a significant difference was observed between the groups. Previously, clinical varicocele has been reported as a possible cause of male hypogonadism [49], whereas surgical treatment may increase serum testosterone levels in men regardless of age; however, patients with near baseline values seem to enjoy the greatest benefit [50]. In our present study, we did not find any dramatic impact of SV on serum testosterone, although patients with abnormal semen parameters had lower levels, a finding that may indicate a cumulative effect of SV on testicular function.

Some aspects regarding our present study should be taken into consideration. Firstly, we did not compare our present results with a control arm of healthy volunteers, as there are ethical concerns regarding the request for semen parameters. Secondly, we evaluated the fertility potential based on the semen parameters only and not the childbearing status of the subjects, which might more specifically reflect fertility, and we did not investigate the impact of varicocelectomy, which could elucidate a treatment effect. Furthermore, conducting the study may raise concerns regarding its justification, as far as the treatment of the unilateral, left SV is not recommended by current guidelines [12]. However, various reports remind us that the condition should not be overlooked. For instance, a SV may play a pivotal role in fertility status when accompanied by a left clinical one, as already mentioned [10]. Additionally, infertile patients with SV may enjoy improvements in their semen quality after correction, which may permit the use of less demanding assisted-reproduction technologies; such results are consistent with the beneficial impact of treatment of clinical varicoceles [14]. Moreover, small or SVs or collaterals, which may become clinically apparent in the future may be the cause of the recurrence of clinical forms [51]. Therefore, we believe that the results of our present study have reasserted the significance of the condition, whereas the selection of CDU, as a daily tool in urological practice, attaches reproducibility to our results. In addition, specific observations such as bilateral disease or the use of haemodynamic markers may prove helpful to the urologist in order to map the management of patients with SV who are at risk or suffer from infertility.

In conclusion, our present results showed that classic US signs, such as venous size, TTV alone or type of reflux were not sufficient to indicate patients with SV and dyspermia. On the other hand, men with abnormal semen parameters and SV have significant differences in their testicular haemodynamics; findings that implicate that impaired testicular microcirculation may be a cause of dyspermia. Furthermore, it seems that the presence of bilateral SV increases the risk of abnormalities in the sperm; whether these men could benefit or not by surgical intervention could be a future challenge. Finally, patients with SV and semen abnormalities had lower, albeit within normal limits, testosterone, findings that indicate a possible negative effect of SV on testicular, endocrine function.
